# Supplementary material for: Distinct spatiotemporal patterns of cortical thinning in Alzheimer’s disease-type cognitive impairment and subcortical vascular cognitive impairment
Source: Commun Biol. 2024 Feb 17;7:198. doi: 10.1038/s42003-024-05787-5 (PMC10874406; doi:10.1038/s42003-024-05787-5)
Supplement: Supplementary file 2 — Supplementary Information [file 42003_2024_5787_MOESM2_ESM.pdf]

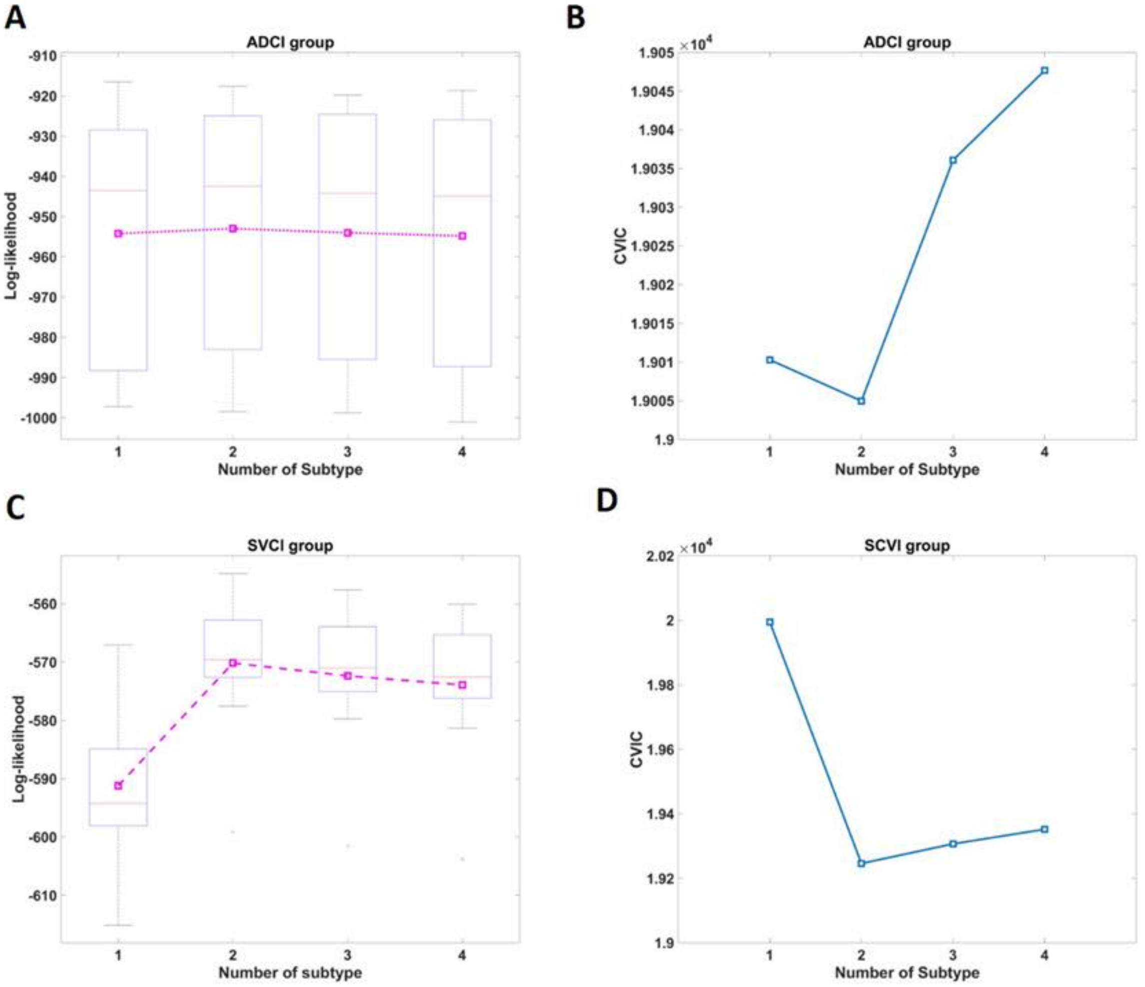

Supplementary Fig 1. Plots of log-likelihood and cross-validation information criterion (CVIC) with respect to different numbers of subtypes. The top row is for the ADCI patients and the bottom row is for the SCVI patients. Using two subtypes was optimal due to the highest log-likelihood and lowest CVIC values for SCVI patients. Magenta line indicates mean value for log-likelihood for 10-fold cross-validation. For ADCI patients, using two subtypes had slightly higher log-likelihood (mean value -952.56) than using just one subtype (mean value -954.20) and using two subtypes had the lowest CVIC.

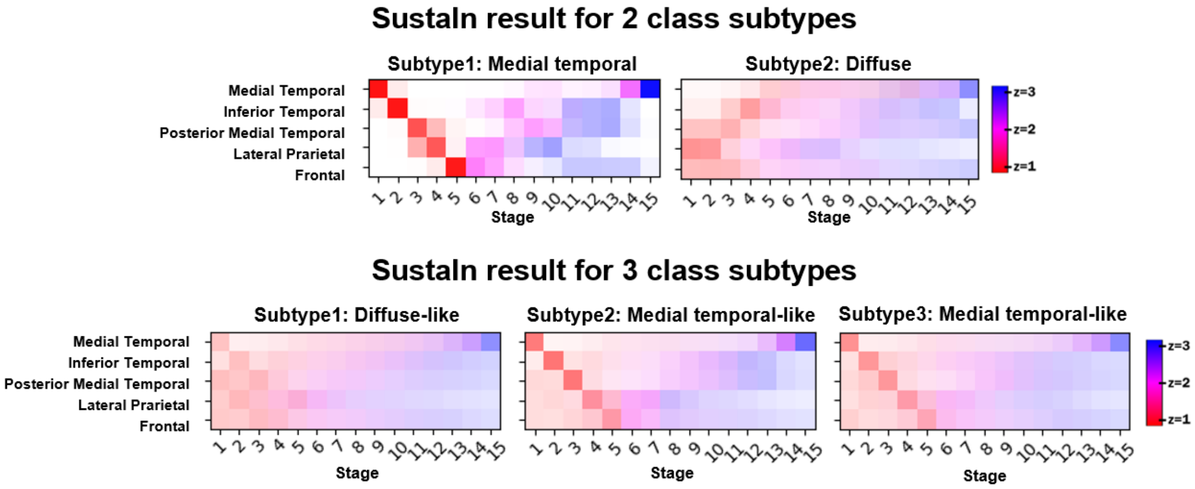

Supplementary Fig 2. Comparison biomarkers for using two and three subtypes for the ADCI patients. The medial temporal subtype among the 2 class subtypes corresponded to subtypes 2 and 3 in the 3 class subtypes, while the diffusion subtype among the 2 class subtypes matched with subtype 1 in the 3 class subtypes.

Supplementary Table 1. Comparison of subtypes between using two and three clusters for the ADCI patients.

Group comparison for SustaIn subtypes	Medial temporal subtypes 2 class subtype1 n = 469	Medial temporal subtypes 3 class subtype2 n = 234	Medial temporal subtypes 3 class subtype3 n = 187	*p*	Diffuse subtypes 2 class subtype2 n = 244	Diffuse subtypes 3 class subtype1 n = 292	*p*
**Demographics**							
Age	70.3±9.4	71.0±9.4	68.9±8.9	0.090†	71.7 ± 9.2	70.9±8.8	0.738†
Female sex (n (%))	227 (59.1%)	129 (55.1%)	99 (52.9%)	0.305†	127 (52.1%)	176 (60.3%)	0.056†
Education (years)	11.6±4.6	11.6±4.9	11.7±4.6	0.983†	11.8±4.8	11.7±4.5	0.906†
APOE genotypes				0.832†			0.674†
E3 homozygotes	186 (39.7%)	91 (38.9%)	71 (38.0%)		95 (38.9%)	91 (40.8%)	
E2 carrier	13 (2.8%)	6 (2.6%)	2 (1.1%)		8 (3.3%)	6 (4.4%)	
E3 carrier	270 (57.6%)	137 (58.5%)	114 (60.9%)		141 (57.8%)	137 (54.8%)	
**Cognitive profiles**							
K-MMSE	21.4±6.2	22.7±5.6	22.5±6.1	1.000‡	22.5±6.1	22.0±5.6	0.568‡
SNSB-II	(n=366)	(n=183)	(n=138)		(n=188)	(n=223)	
Digit span backward	3.5±1.4	3.6±1.4	3.4±1.5	1.000‡	3.4±1.2	3.1±1.2	1.000‡
K-BNT	37.9±13.3	38.6±12.5	37.1±15.0	1.000‡	38.9±13.0	37.7±13.1	1.000‡
RCFT-copy	25.7±10.5	26.5±10.2	24.2±11.6	1.000‡	24.7±11.3	24.4±10.9	1.000‡
SVLT-delayed recall	1.3±2.1	1.4±2.3	1.9±2.6	0.206‡	2.0±2.7	1.4±2.2	0.016‡
RCFT-delayed recall	4.1±5.3	4.2±5.4	5.8±6.5	0.094‡	5.7±6.4	4.0±5.2	0.040‡
COWAT-Semantic	21.8±10.1	21.6±10.2	23.0±11.5	1.000‡	23.2±9.9	20.9±9.3	0.544‡
Stroop color reading	52.0±32.5	56.7±32.5	54.0±32.5	1.000‡	51.7±33.2	47.3±31.5	0.360‡

†The p values were adjusted using Bonferroni's method because of inflated type I error.

‡The p values were obtained by analysis of covariance after controlling for age, sex, and education level, and adjusted using Bonferroni's method because of inflated type I error.

ADCI = Alzheimer's disease-type cognitive impairment; APOE = Apolipoprotein; K-MMSE = Korean version of the Mini-Mental State Examination; SNSB-II = Seoul Neuropsychological Screening Battery 2nd edition; K-BNT = Korean version of the Boston Naming Test; RCFT = Rey–Osterrieth Complex Figure Test; SVLT = Seoul Verbal Learning Test; COWAT = Controlled Oral Word Association Test

Supplementary Table 2. Baseline demographic variables and cognitive profiles of each SuStaIn subtypes using three clusters in ADCI patients.

ADCI (n = 713)	Diffuse-like Subtype 1 (n=292)	Medial temporal-like Subtype 2 (n = 234)	Medial temporal-like Subtype 3 (n = 187)	*p*	post hoc anaylsis
**Demographics**					
Age	71.2±9.2	71.4±9.6	69.2±9.0	0.108†	
Sex, female (n (%))	176 (60.3)	129 (55.1)	99 (52.9)	0.729†	
Education (years)	71.2±9.2	71.4±9.6	69.2±9.0	1.000†	
APOE genotype				0.492†	
E3 homozygotes	121 (41.4)	91 (38.9)	69 (36.9)		
E2 carrier	13 (4.5)	6 (2.6)	2 (1.1)		
E4 carrier	158 (54.1)	137 (58.6)	116 (62.0)		
**Cognitive profiles**					
K-MMSE	21.7±5.9	22.0±6.1	21.7±6.9	0.800‡	
SNSB-II	(n=227)	(n = 220)	(n = 177)		
Digit span backward	3.1±1.2	3.5±1.5	3.3±1.5	0.011‡	2>1
K-BNT	37.2±13.2	37.5±13.6	35.3±16.3	0.074‡	
RCFT-copy	24.0±11.1	26.0±10.5	23.4±12.1	0.056‡	
SVLT-delayed recall	1.3±2.2	1.4±2.3	1.8±2.5	0.054‡	
RCFT-delayed recall	3.9±5.1	4.0±5.3	5.3±6.4	0.011‡	
COWAT-Semantic	20.8±9.5	21.4±10.3	22.8±11.5	0.169‡	
Stroop color reading	44.9±31.7	53.3±34.0	48.9±34.6	0.008‡	2>1

†The p values were adjusted using Bonferroni's method because of inflated type I error.

‡The p values were obtained by analysis of covariance after controlling for age, sex, and education level, and adjusted using Bonferroni's method because of inflated type I error.

ADCI = Alzheimer's disease-type cognitive impairment; APOE = Apolipoprotein; K-MMSE = Korean version of the Mini-Mental State Examination; SNSB-II = Seoul Neuropsychological Screening Battery 2nd edition; K-BNT = Korean version of the Boston Naming Test; RCFT = Rey–Osterrieth Complex Figure Test; SVLT = Seoul Verbal Learning Test; COWAT = Controlled Oral Word Association Test

Supplementary Table 3. Mixed effects models analyzing the relationship between SuStaIn subtypes using three clusters and rate of cognitive decline in ADCI patients

Cognition parameter	ADCI subtype × time		post hoc analysis
	β (S.E)	*p* for interaction	
**K-MMSE**			
2 vs 1	-0.349 (0.07)	< 0.001†	
3 vs 1	-0.536 (0.09)	< 0.001†	2=3>1
3 vs 2	-0.185 (0.09)	0.984†	
**SNSB-II**			
Digit span backward			
2 vs 1	-0.077 (0.02)	< 0.001†	
3 vs 1	-0.065 (0.03)	0.408†	2>1
3 vs 2	0.028 (0.03)	1.000†	
K-BNT			
2 vs 1	-0.593 (0.14)	< 0.001†	
3 vs 1	-0.743 (0.18)	< 0.001†	2=3>1
3 vs 2	0.023 (0.21)	1.000†	
RCFT-copy			
2 vs 1	-0.179 (0.13)	1.000†	
3 vs 1	-0.374 (0.17)	0.648†	
3 vs 2	-0.136 (0.18)	1.000†	
SVLT-delayed recall			
2 vs 1	-0.103 (0.04)	0.264†	
3 vs 1	-0.030 (0.05)	1.000†	
3 vs 2	0.068 (0.06)	1.000†	
RCFT-delayed recall			
2 vs 1	-0.250 (0.09)	1.000†	
3 vs 1	-0.166 (0.12)	1.000†	
3 vs 2	0.127 (0.13)	1.000†	
COWAT-Semantic			
2 vs 1	-0.486 (0.16)	0.048†	
3 vs 1	-0.904 (0.20)	< 0.001†	2=3>1
3 vs 2	-0.333 (0.22)	1.000†	
Stroop color reading			
2 vs 1	-0.702 (0.41)	1.000†	
3 vs 1	-1.275 (0.51)	0.312†	
3 vs 2	-0.386 (0.57)	1.000†	

Model: fixed effect: age, sex, education level, SuStaIn subtype, baseline cognition, time interval from initial evaluation (years), sustain subtype × time interval; random effect: subject.

†p values were adjusted using Bonferroni's method because of inflated type I error.

Three subtypes were indicated as follows: 1, diffuse-like subtype; 2 and 3, medial temporal-like subtypes.

ADCI = Alzheimer's disease-type cognitive impairment; APOE = Apolipoprotein; K-MMSE = Korean version of the Mini-Mental State Examination; SNSB-II = Seoul Neuropsychological Screening Battery 2nd edition; K-BNT = Korean version of the Boston Naming Test; RCFT = Rey–Osterrieth Complex Figure Test; SVLT = Seoul Verbal Learning Test; COWAT = Controlled Oral Word Association Test
