# Supplementary material for: Distinct spatiotemporal patterns of cortical thinning in Alzheimer’s disease-type cognitive impairment and subcortical vascular cognitive impairment
Source: Commun Biol. 2024 Feb 17;7:198. doi: 10.1038/s42003-024-05787-5 (PMC10874406; doi:10.1038/s42003-024-05787-5)
Supplement: Supplementary file 3 — Description of Additional Supplementary Files [file 42003_2024_5787_MOESM3_ESM.pdf]

Description of Additional Supplementary Files

File name: Supplementary Data 1

Description: The source data of the main figures
